# Native Pulmonic Valve Endocarditis due to *Mycobacterium fortuitum*: A Case Report and Literature Review

**DOI:** 10.1155/2015/274819

**Published:** 2015-06-08

**Authors:** Aaron M. Mulhall, Renee S. Hebbeler-Clark

**Affiliations:** ^1^Division of Pulmonary, Critical Care, and Sleep Medicine, University of Cincinnati Medical Center, 231 Albert Sabin Way, Cincinnati, OH 45267, USA; ^2^Division of Infectious Diseases, University of Cincinnati Medical Center, 231 Albert Sabin Way, Cincinnati, OH 45267, USA

## Abstract

Endocarditis secondary to *Mycobacterium fortuitum* is a rare entity often involving prosthetic valves and rarely native valves. Pulmonic valve endocarditis secondary to any organism is rare. We report the first case of native pulmonic valve endocarditis secondary to *M. fortuitum* and a literature review of native valve *M. fortuitum* endocarditis.

## 1. Introduction

*Mycobacterium fortuitum*, a member of rapidly growing nontuberculous mycobacteria, is a well-known cause of skin and soft tissue infections and postsurgical wound infections [1]. Sporadic cases of endocarditis associated with this organism have been reported, mostly involving prosthetic valves. Isolated pulmonic valve endocarditis caused by any organism is rare, making up about 2% of all cases of infective endocarditis [2]. Nontuberculous mycobacteria pulmonic valve endocarditis has never been reported in the literature. Here, we describe a case of native pulmonic valve endocarditis secondary to* M. fortuitum* as well as a review of the literature.

## 2. Case Presentation

**Figure 1:**
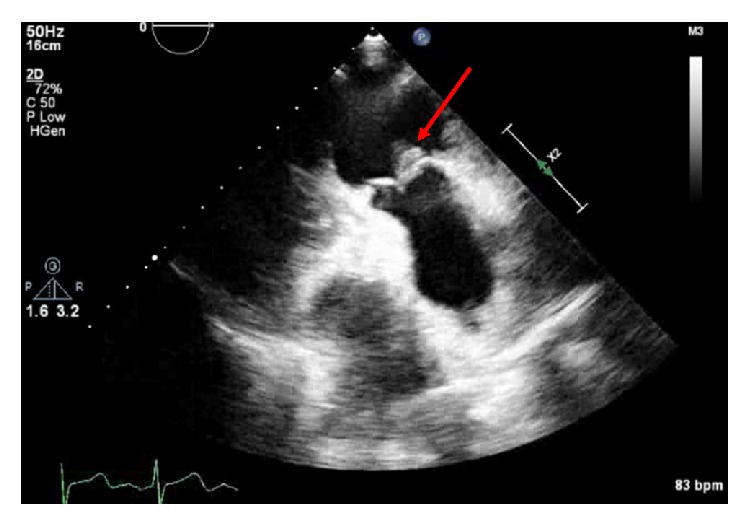
Transthoracic echocardiogram showing a 10 mm × 11 mm vegetation (arrow) on the ventricular aspect of the pulmonic valve.

A 64-year-old female with a history of ventilator dependent respiratory failure secondary to end-stage chronic obstructive pulmonary disease (COPD) and bioprosthetic mitral valve replacement 8 years ago was admitted to the medical intensive care unit (MICU) for worsening respiratory failure from pulmonary edema and multifocal pneumonia secondary to* Escherichia coli*. She was diuresed and completed a course of antibiotics with ceftriaxone. A transesophageal echocardiogram (TEE) was performed during this admission revealing severe prosthetic mitral valve stenosis, mildly elevated pulmonary artery pressures, a normal tricuspid valve, and no vegetation on any valve. She was ultimately transferred to a long term postacute care hospital. Over the following month, the patient continued to decline in clinical status, and further investigation was undertaken. Rapid-growing mycobacterium was isolated from three cultures for mycobacteria from a tracheal aspirate. Interferon Gamma Release Assay (IGRA) and* M. tuberculosis* complex polymerase chain reaction (PCR) of a tracheal aspirate were negative. Rapid-growing mycobacterium was isolated from multiple blood cultures from a peripherally inserted central catheter (PICC) and from peripheral venipuncture.* M. fortuitum* was the organism identified in all cultures. Empiric antibiotic therapy was initiated with amikacin, imipenem, and clarithromycin; and the PICC line was removed. The resistance-pattern confirmed that the isolate was sensitive to amikacin and imipenem, but resistant to clarithromycin. The patient was ultimately readmitted to the MICU for hypotension and concerns for overdiuresis and volume depletion. A transthoracic echocardiogram (TTE) was performed (40 days after the previous TTE and 12 days after initiation of antibiotics) revealing a new 10 mm × 11 mm vegetation on the ventricular aspect of the pulmonic valve, severe tricuspid regurgitation, and severe prosthetic valve mitral stenosis (Figure 1). The patient was not a candidate for pulmonic valve replacement and medical management was continued. Given the grim prognosis, the patient ultimately decided to stop antibiotic therapy and entered hospice where she ultimately passed away. The patient completed 16 days of antibiotics at the time she was discharged to hospice.

## 3. Discussion

*Mycobacterium fortuitum* is a ubiquitous organism that can be found in soil, dust, and tap water and is well known for causing skin infections and postsurgical wound infections [1]. It belongs to the family of rapid-growing nontuberculous mycobacteria, meaning that it grows in culture within one week. Infections with this organism are often a result of direct inoculation, usually as a result of posttraumatic infections, postsurgical wound infections, and catheter-associated infections. Disseminated infections mostly occur in immunodeficient patients [3].

Endocarditis caused by this organism is uncommon, mostly affecting patients with prosthetic valves [4–6]. Endocarditis of native cardiac valves due to* M. fortuitum* is a rare occurrence, with only a small number of cases being reported [7–12] (Table 1). Upon review of the nine cases of native valve* M. fortuitum* endocarditis (including our own) the mean age was 24.4 years old (range 0.5–64 years); the population was 66% female; valves infected were 44% aortic, 44% tricuspid (one patient with both aortic and tricuspid vegetation), 22% mitral (one patient with both aortic and mitral vegetation), and 11% pulmonic; 56% had either an open or percutaneous cardiac procedure as the inciting event inoculating the patient and 22% were infected secondary to intravenous drug use; 22% received a valve replacement or removal of implanted cardiac hardware; and 44% of patients were alive at the time of their respective case report publication (in our case, the patient entered hospice).

There is only anecdotal evidence for the recommended treatment of severe disseminated* M. fortuitum* infections. All reviewed cases of native valve endocarditis secondary to this organism were treated with parenteral amikacin plus two of the following: fluoroquinolone, cefoxitin, TMP/SMX, or a macrolide. These agents should be initiated and continued for several weeks (most cases were treated for 6 weeks) until clinical improvement was identified and then transitioned to oral therapy with two effective agents for 6–12 months.

Our patient's isolate was sensitive to amikacin and imipenem, but resistant to clarithromycin. It is difficult to determine if the vegetation arose during antibiotic therapy, as she was only treated for 12 days prior to finding this vegetation. Antibiotic resistance and bacterial control were not major concerns based on the isolate resistance-pattern. We believe that she likely developed this vegetation in the previous month when she was clinically declining from an unclear etiology. Unfortunately, in this case, we could not confirm, by tissue diagnosis, that the vegetation was due to* M. fortuitum*. This is because the patient was not a surgical candidate for pulmonic valve replacement and the family declined a postmortem examination. While it is possible that the vegetation was not due to* M. fortuitum*, it is highly unlikely in the setting of new cardiac valve vegetation and persistently positive blood cultures for* M. fortuitum*.

There are a couple of potential mechanisms to explain how our patient became infected. One possibility is that our patient had airway colonization with* M. fortuitum* related to her end-stage COPD and the organism entered her bloodstream, subsequently seeding her pulmonic valve.* M. fortuitum* is commonly isolated from respiratory cultures in patients with underlying lung disease including COPD, pulmonary fibrosis, bronchiectasis, and prior pulmonary tuberculosis infection [13].

It is thought that the mycobacteria in the lungs gains access to the bloodstream via translocation. Previous studies of* M. tuberculosis* suggest that the organism is ingested by alveolar macrophages and then is transported by these macrophages and peripheral blood monocytes across the alveolar wall into the bloodstream. More recent evidence suggests that mycobacteria can also invade type II alveolar epithelial cells and translocate across the epithelial wall into the bloodstream [14]. Another potential mechanism is that our patient acquired* M. fortuitum* by a catheter-associated bloodstream infection that subsequently seeded her lungs and pulmonic valve. The propensity of* M. fortuitum* to cause catheter-related infections, its resistance to a number of antibiotics, and its association with tap water and water distribution centers have some relation with its ability to form biofilms [15].

Our case is the first reported case of* M. fortuitum* native valve endocarditis affecting the pulmonic valve. It is also the first reported case to have the organism isolated from a pulmonary source (tracheal aspirate) as well as the bloodstream. Even with antibiotics this disease is highly fatal and source control with valve replacement/removal of infectious hardware greatly improves survival [12]. The extremely poor prognosis associated with this disease reinforces the need to consider endocarditis in any patient with* Mycobacterium* bacteremia, regardless of species.

## Figures and Tables

**Table 1:** Reported cases of native valve endocarditis caused by *Mycobacterium fortuitum*.

Reference	Age	Gender	Valves affected	Associated procedures	Comorbidities	Organism isolated from other sources	Surgical therapy	Antibiotic therapy	Survival
Our patient (2015)	64	Female	Pulmonic	None	End-stage COPD, bioprosthetic MVR, mitral stenosis	Tracheal aspirate, PICC	No	Amikacin, imipenem, clarithromycin	No

Vuković et al. [7]	12	Female	Tricuspid	Bovine patch VSD repair	None	No	No	Not reported	Alive at 12 months after diagnosis
	2	Female	Tricuspid	Bovine patch VSD repair	Down syndrome	No	No	Not reported	Alive at 12 months after diagnosis
	0.5	Male	Tricuspid	Bovine patch VSD repair	None	Vegetation, VSD patch	VSD patch removal, VSD repair	Not reported	Alive at 12 months after diagnosis

Natsag et al. [8]	49	Female	Aortic, tricuspid	IVDU	Skin abscesses, hepatitis C	No	No	TMP/SMX, linezolid, ciprofloxacin	Alive at 6 weeks after diagnosis

Collison and Trehan [9]	50	Male	Mitral, aortic	PCI and stent placement	Coronary artery disease, congestive heart failure	Not reported	AVR, MVR, coronary artery bypass graft	Clarithromycin, imipenem, moxifloxacin, amikacin	No

Singh et al. [10]	54	Female	Aortic	Hemodialysis	Aortic stenosis, mitral regurgitation, ESRD	No	No	TMP/SMX, ciprofloxacin, amikacin, clofazimine	No

Spell et al. [11]	47	Male	Aortic	IVDU	Human immunodeficiency virus	No	AVR offered (patient declined)	Amikacin, ciprofloxacin, cefoxitin	No

Kuruvila et al. [12]	20	Female	Mitral	Balloon mitral valvulotomy	Rheumatic heart disease	Cerebrospinal fluid	No	Amikacin, azithromycin, rifampin	No

VSD: ventricular septal defect; PCI: percutaneous coronary intervention; IVDU: intravenous drug use; COPD: chronic obstructive pulmonary disease; ESRD: end-stage renal disease; PICC: peripherally inserted central catheter; MVR: mitral valve replacement.
